# The complete chloroplast genome sequence of *Thunbergia erecta* (Benth.) *T. Anders*. (Acanthaceae)

**DOI:** 10.1080/23802359.2022.2140574

**Published:** 2022-11-15

**Authors:** Lili Tong, Lu Tian, Xiaogang Xu, Yao Cheng

**Affiliations:** a School of Horticulture & Landscape Architecture, Jinling Institute of Technology, Nanjing, China; b Co-Innovation Center for Sustainable Forestry in Southern China, College of Biology and the Environment, Key Laboratory of State Forestry and Grassland Administration on Subtropical Forest Biodiversity Conservation, Nanjing Forestry University, Nanjing, China; c State Environmental Protection Scientific Observation and Research Station for Ecology and Environment of Wuyi Mountains, Nanping, China

**Keywords:** *Thunbergia erecta*, phylogenetic, Acanthaceae, complete chloroplast genome

## Abstract

*Thunbergia erecta* (Benth.) *T. Anders*. is an upright shrub species of Acanthaceae with great ecological and economical values. In this paper, we explored the complete chloroplast genome sequence of *T. erecta* using next generation sequencing to provide genomic resources that could help to promote its conservation. The genome of *T. erecta* is 152,202 bp in length, containing a large single-copy region of 84,232 bp, and a small single-copy region of 17,656 bp. It encodes a total of 131 genes, including 8 rRNA genes, 37 tRNA genes, 84 protein-coding genes and 2 pseudo genes. The GC content of *T. erecta* genome is 38.47%. The phylogenetic analysis suggests that it was closely related to *Avicennia marina* in Acanthaceae. In addition, we found that *T. erecta* appeared late clade in the whole family while outgroups plants appeared even later. *T. erecta* is morphologically similar to other plants in Acanthaceae, but is genetically closed to the outgroup species.

*Thunbergia erecta* is a prominent species of Acanthaceae, around two meters tall, which is native to tropical West Africa and cultivated as ornamental plants around the world (Hu and Tsui 2002, 2011). It possesses high value for medicinal (Refaey et al. 2021) and ornamental purposes and a good vertical greening material for scaffolds, flower fences and walls as well. However, little progress has been made to its complete chloroplast (cp) genome. In this work, we characterized the complete cp genome sequence of *T. erecta*, based on Illumina pair-end sequencing data, and deposited the sequence in GeneBank (MZ555773) to provide a valuable complete cp genome resource.

The fresh leaves were collected from Xishuangbanna Tropical Botanical Garden (**latitude** 21.6840 and **longitude** 101.4677) in Jinghong, Yunnan, China. A specimen was deposited at the herbarium of Nanjing Forestry University (contact person: Xuehong Ma; E-mail: xuehongma@njfu.edu.cn) under the voucher number NF2021038. According to the International Union for Conservation of Nature (IUCN) policy on endangered species research, the sample collection and the study was conducted with permission from Xishuangbanna Tropical Botanical Garden. The genomic DNA was extracted and then sequenced based on Illumina pair-end sequencing data. By applying ultrasound to break DNA, the fragments of DNA were passivated, repaired and bonded and selected by agarose gel electrophoresis. The sample of genome sequencing library was formed by PCR amplification, which was carried out on Illumina Novaseq platform with PE150 reads by Nanjing Genepioneer Biotechnologies Inc. (Nanjing, China).

The original reading was filtered by fastp (version 0.20.0), and the clean data were assembled into chloroplast genome using SPAdes (Bankevich et al. 2012). There were no uncertain bases in the assembly results. Then, the reference sequence (Genebank accession number: NC050991) was used for quality control after assembly, and the assembled genome was annotated using CpGAVAS2 (Shi et al. 2019). The complete cp genome sequences of species were acquired necessary from NCBI.

The complete cp genome of *T. erecta* was 152,202 bp in length, containing a large single-copy region (LSC) of 84,232 bp, a small single-copy region (SSC) of 17,656 bp, and a pair of inverted repeat regions (IRs) of 25,157 bp. The new sequence possesses 131 genes, including 84 protein-coding genes (78 CDS species), 37 tRNA genes (30 tRNA species), 8 rRNA genes (4 rRNA species) and 2 pseudo genes. Among them, 6 protein-coding genes (*ndhB*, *rpl2*, *rpl23*, *rps12*, *rps7* and *ycf2*), 7 tRNA genes (*trnA*-*UGC*, *trnI*-*CAU*, *trnI*-*GAU*, *trnL*-*CAA*, *trnN*-*GUU*, *trnR*-*ACG* and *trnV*-*GAC*), and 4 distinct rRNA genes (*4.5S*, *5S*, *16S* and *23S*) were duplicated, apart from that, most of genes occurred in a single copy. A total of 10 protein-coding genes (*atpF*, *ndhA*, *ndhB*, *petB*, *petD*, *rpl16*, *rpl2*, *rpoC1*, *rps12* and *rps16*) contained 1 intron while the other 2 genes (*clpP*, *ycf3*) had 2 introns each. The overall GC content of cp genome was 38.47%, while the corresponding values of the LSC, SSC and IR regions were 36.57%, 33.15% and 43.52%, respectively (Figure 1).

**Figure 1.**
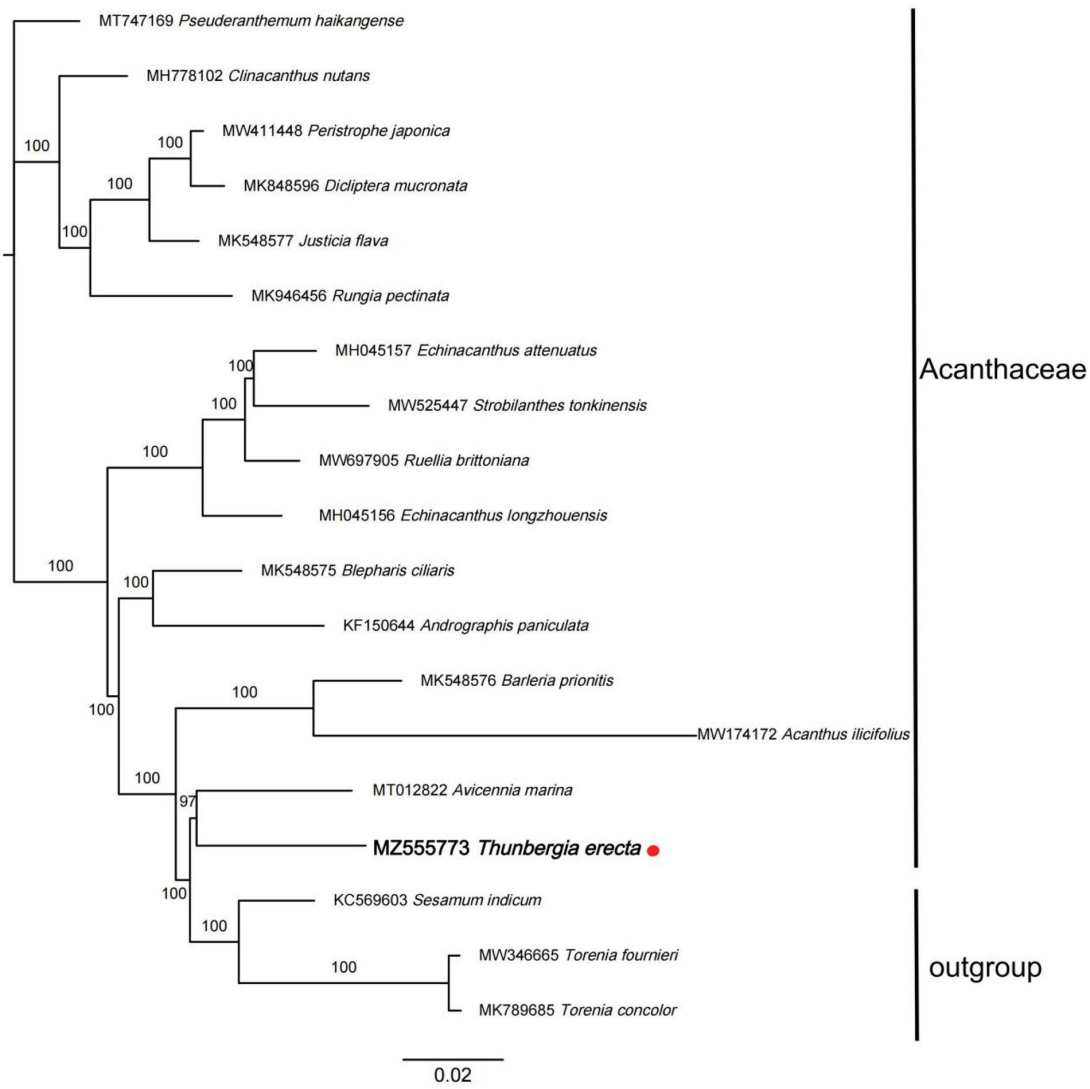
Maximum Likelihood tree showing the relationship among *Thunbergia erecta* and representative species within Acanthaceae, based on whole chloroplast genome sequences, with 2 taxa from Lamiales as outgroup. The bootstrap supports the values shown at the branches.

To reveal the phylogenetic evolution of *T. erecta*, we constructed a ML phylogenetic tree based on 16 cp genomes from Acanthaceae and 3 cp genomes as outgroups from 2 taxa (Pedaliaceae, Linderniaceae). The sequence aligment by MAFFT (Rozewicki et al. 2019), IQTREE (Garg and Biju 2021) was used to perform maximum Likelihood (ML) tree with the TVM + F+R4 model. The bootstrap method was used to test the reliability of phylogeny with 1000 replicates.

The phylogenetic analysis suggests that *T. erecta* was closely related to *Avicennia marina* in Acanthaceae. In addition, *T. erecta* was more likely appeared in late differentiation stage in the whole family succession while outgroups species did even later. *T. erecta* is morphologically similar to other species in Acanthaceae, but is genetically closed to those in outgroup. As the increasing of sample collection sequences of this taxa and the deepening of research, its status will become clearer.

## Data Availability

The genome sequence data that support the findings of this study are openly available in GenBank of NCBI at [https://www.ncbi.nlm.nih.gov] (https://www.ncbi.nlm.nih.gov/) under the accession no. MZ555773. The associated BioProject, SRA, and Bio-Sample numbers are PRJNA745545, SRR15100132, and SAMN20169050 respectively.
